# Molecular Recognition of FDA-Approved Small Molecule Protein Kinase Drugs in Protein Kinases

**DOI:** 10.3390/molecules27207124

**Published:** 2022-10-21

**Authors:** Yan Zhu, Xiche Hu

**Affiliations:** Department of Chemistry and Biochemistry, University of Toledo, Toledo, OH 43606, USA

**Keywords:** FDA-approved drugs, molecular recognition, quantum chemical calculations, π-π stacking interactions, CH-π interactions, rational drug design

## Abstract

Protein kinases are key enzymes that catalyze the covalent phosphorylation of substrates via the transfer of the γ-phosphate of ATP, playing a crucial role in cellular proliferation, differentiation, and various cell regulatory processes. Due to their pivotal cellular role, the aberrant function of kinases has been associated with cancers and many other diseases. Consequently, competitive inhibition of the ATP binding site of protein kinases has emerged as an effective means of curing these diseases. Decades of intense development of protein kinase inhibitors (PKIs) resulted in 71 FDA-approved PKI drugs that target dozens of protein kinases for the treatment of various diseases. How do FDA-approved protein kinase inhibitor PKI drugs compete with ATP in their own binding pocket? This is the central question we attempt to address in this work. Based on modes of non-bonded interactions and their calculated interaction strengths by means of the advanced double hybrid DFT method B2PLYP, the molecular recognition of PKI drugs in the ATP-binding pockets was systematically analyzed. It was found that (1) all the FDA-approved PKI drugs studied here form one or more hydrogen bond(s) with the backbone amide N, O atoms in the hinge region of the ATP binding site, mimicking the adenine base; (2) all the FDA-approved PKI drugs feature two or more aromatic rings. The latter reach far and deep into the hydrophobic regions I and II, forming multiple CH-π interactions with aliphatic residues L(3), V(11), A(15), V(36), G(51), L(77) and π-π stacking interactions with aromatic residues F(47) and F(82), but ATP itself does not utilize these regions extensively; (3) all FDA-approved PKI drugs studied here have one thing in common, i.e., they frequently formed non-bonded interactions with a total of 12 residues L(3),V(11), A(15), K(17), E(24),V(36),T(45), F(47), G(51), L(77), D(81) and F(82) in the ATP binding. Many of those 12 commonly involved residues are highly conserved residues with important structural and catalytic functional roles. K(17) and E(24) are the two highly conserved residues crucial for the catalytic function of kinases. D(81) and F(82) belong to the DFG motif; T(45) was dubbed the gate keeper residue. F(47) is located on the hinge region and G(51) sits on the linker that connects the hinge to the αD-helix. It is this targeting of highly conserved residues in protein kinases that led to promiscuous PKI drugs that lack selectivity. Although the formation of hydrogen bond(s) with the backbone of the hinge gives PKI drugs the added binding affinity and the much-needed directionality, selectivity is sacrificed. That is why so many FDA-approved PKI drugs are known to have multiple targets. Moreover, off-target-mediated toxicity caused by a lack of selectivity was one of the major challenges facing the PKI drug discovery community. This work suggests a road map for future PKI drug design, i.e., targeting non-conserved residues in the ATP binding pocket to gain better selectivity so as to avoid off-target-mediated toxicity.

## 1. Introduction

Protein kinases are key enzymes that catalyze the covalent phosphorylation of substrates via transfer of the γ-phosphate of ATP [1,2], functioning as switches to “turn on” or “turn off” the substrate proteins. It has been estimated that the human genome encodes 538 known protein kinase genes [3,4], which are nearly 2% of all genes. They play a pivotal role in signal transduction and regulation of cellular functions, such as cellular proliferation, differentiation, and necrosis [5,6,7,8,9]. Protein kinases are classified into two types based on the phosphorylated amino acids of their substrates: serine/threonine kinases (STK) that act on serine or threonine, tyrosine kinases (TK) that act on tyrosine [10,11]. Due to their pivotal role [9], aberrant functions of protein kinases were known to cause many common diseases such as cancer, immunodeficiency, diabetes, atherosclerosis, and psoriasis [1,12,13,14,15,16]. Therefore, the inhibition of aberrant protein kinases has the therapeutic potential to cure these diseases [1,17,18]. Over the years, the discovery of protein kinase inhibitor drugs (PKI drugs) has emerged as a subject of great theoretical importance and therapeutic value [19,20].

Despite their diverse primary structure organizations, the catalytic domains of various kinases are generally conserved [9]. It consists of two lobes: the small N-lobe is dominated by anti-parallel β-sheet, and the large C-lobe is primarily formed of α-helices. The nucleotide, ATP, binds at the base of the cleft between the two lobes, positioning the γ-phosphate for transfer to the peptide substrate that binds to the surface of the large C-lobe. Another important structural feature is the DFG (Asp-Phe-Gly) motif, which is a highly conserved sequence located at the beginning of the activation loop [21,22]. The motif’s Asp residue is responsible for coordinating a magnesium ion which positions the phosphates of ATP for phosphotransfer. The Phe residue of the motif packs under the helix C (αC-helix) and is therefore important for the correct positioning of this helix. It is important to note that the αC-helix itself acts as a dynamic regulatory element for the catalytic function of protein kinases. In the active state of kinases, the DFG motif adopts an “in” conformation, with the Asp residue properly oriented toward bound ATP for transferring of the γ-phosphate group to the substrate. In the inactive state of kinases, the DFG motif flips outward to adopt an “out” conformation in which the Phe residue of the DFG motif moves more than 10 Å from its position in the active kinase conformation [21,22]. As a result, the Asp residue no longer coordinates the magnesium at the catalytic site, rendering the kinase inactive. 

Based on their binding modes with targeted protein kinases, small molecule PKIs can be classified into Types 1, 2, and 3 inhibitors [23,24,25,26]. Type 1 inhibitor is defined as a small molecule that binds to the active conformation of a kinase in the ATP pocket; the Type 2 inhibitor binds to an inactive (usually DFG-OUT) conformation of a kinase; and the Type 3 inhibitor binds next to the ATP-binding pocket allosterically, and is a non-ATP competitive inhibitor. In this study, the scope is limited to ATP competitive inhibitors only. 

**Figure 1:**
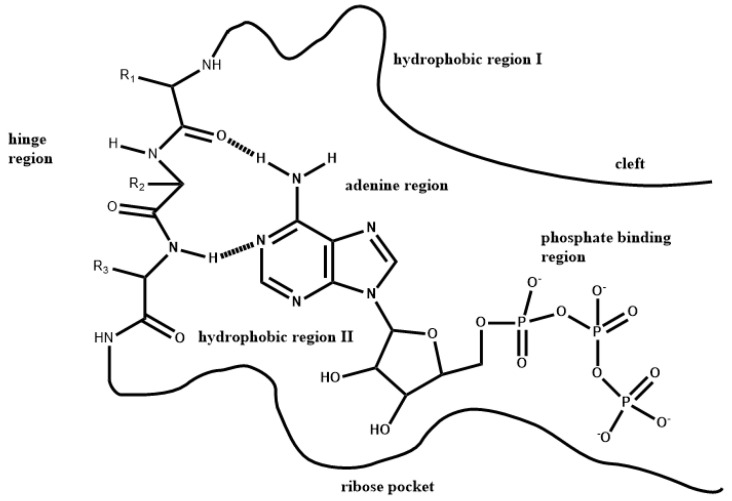
Pharmacophore model for Type 1 PKI drugs in the ATP binding pocket of protein kinases [28].

The Type 1 PKIs typically bind to the DFG-in active (open) conformation of protein kinases, mimicking the interactions of the adenine moiety of ATP [27] by forming multiple hydrogen bonds with the kinase hinge. Based on structural biology studies, the binding pocket for Type 1 PKIs can be divided into subregions: the hinge region; the adenine region; the hydrophobic region I; the hydrophobic region II; the ribose pocket; and the phosphate-binding region [23,24]. Shown in Figure 1 is a general pharmacophore model for Type 1 PKIs in the ATP binding pocket of protein kinases [25]. Both the ribose pocket and the phosphate-binding region are hydrophilic in nature. The remaining regions are of mostly hydrophobic character [25]. The adenine region is mostly surrounded by hydrophobic residues. The hydrophobic region I extends deeply in the direction of the lone pair of the N7 nitrogen of adenine. The hydrophobic region II corresponds more to a hydrophobic slot opened to the solvent that is not used by ATP. It is worth noting that, although both hydrophobic regions exist naturally, they are not used by ATP in most protein kinases.

In 2001, the first PKI drug imatinib was approved by the FDA for the treatment of chronic myeloid leukemia in the United States, which represented an important milestone in precision medicine [29]. Since then, protein kinases have emerged as one of the most significant drug targets in the 21st century [30]. As of May 2022, a total of 71 FDA-approved small molecule kinase inhibitors are on the market [30]. Furthermore, hundreds of PKI drugs are in different phases of clinical trials worldwide [31], and many kinase-specific inhibitors are in the preclinical stage of drug development [32]. Nevertheless, many challenges remain in PKI drug discovery, including drug resistance and off-target-mediated toxicity [17,32,33,34,35]. The latter resulted from a lack of selectivity. In order to address the issue of drug selectivity, one needs to have a better understanding of the molecular determinants responsible for the molecular recognition of FDA-approved PKI drugs in their respective targeted protein kinases [36]. That is what we aim to do in this work. The central question we attempted to answer here is “How do FDA-approved PKI drugs compete with ATP in its own binding pocket?”

As in all ligand-protein complexes, the molecular recognition between PKI drugs and their target protein kinases are achieved by non-bonded interactions [37,38]. Traditionally, the consideration of non-bonded interactions mainly included hydrogen bonding and salt bridge interactions. However, in recent years, more and more evidence suggests that π-moiety involved interactions, such as π-π interactions [39], CH-π interactions [40], cation-π interactions [41], XH-π interactions (XH=NH, OH, SH), are equally important non-bonded interaction forces [40,42,43,44,45,46,47]. For easy reference, hereinafter, all these π-moiety involved interactions collectively will be named non-bonded π-interactions.

## 2. Results

A large-scale data mining of the PDB databank was carried out, which resulted in a total of 34 distinct non-redundant drug-protein complexes formed by 30 PKI drugs. It is worth noting that some drugs had no reported PDB entries, and several PKI drugs are bound to multiple targets. Provided in Table 1 are molecular structures of the 30 PKI drugs that have available complex structures. All 30 PKI drugs contained two or more aromatic rings. For completeness, Table S1 lists the names of 30 PKI drugs, along with drug company, inhibitor type, year of approval, known targets, and diseases treated. Table 2 lists the PDB IDs for the 34 distinct drug-protein complexes, as well as the drug name, ligand ID for the PKI drug, X-ray crystal structure resolution, and bibliographic reference. Those drug-bound protein kinases belong to 14 kinase families (ABL, ALK, CDK, EGFR, JAKA, MET, PDGFR, RAF, RET, SEV, SRC, STE7, TEC, VEGFR), and span 4 kinase groups (CMGC, STE, TK, TKL) [3,48]. As shown in the table, there are 27 tyrosine kinases and 7 serine/threonine kinases. All 34 drug-protein complexes were superimposed by means of the pairwise 3D structure alignment using the FATCAT algorithm as implemented on the RCSB PDB site [49,50].

Based on the aligned 3D structures, binding pockets of the PKI drugs in their respective target protein kinases were thoroughly examined with the Visual Molecular Dynamic (VMD) program to identify residues that form non-bonded intermolecular interactions with each drug [81]. In accordance with the physical nature of all modes of non-bonded intermolecular interactions, the calculated strengths of the solution phase interaction energies normally die off after 5.5 Å based on our past experience of quantum chemical calculations. Thus, we chose a cutoff distance of 5.5 Å for all modes of intermolecular interactions. Residues within 5.5 Å of any atoms of a given PKI drug were kept as interacting residues for further analysis. 

In the next step, the interacting residues are divided into three classes according to their locations in the drug binding pockets: the hinge region, the front interface and the back interface. The back interface is in the general area around the hydrophobic region I, and the front interface is located around the hydrophobic region II (see Figure 1). In order to describe the two interfaces, it is necessary to familiarize the readers with the nomenclature for the catalytic domain of protein kinases adopted by the KLIFS database [26]. Due to space limitations, only salient features will be briefly highlighted here. At first, a consensus sequence of 85 amino acids in the catalytic cleft was established based on the 3D alignment of 1252 inhibitor kinase complexes; they were numbered from 1 to 85. We will adopt this numbering scheme here, placing the consensus residue ID number in parentheses following the one-letter code for the amino acid. For example, F(82) designates the phenylaniline residue located at the 82nd position of the consensus sequence. Secondly, key elements for the kinase binding site were associated with a sequence number range and named, including the nomenclature for β-sheets I−VIII (I-III); αC-helix (aC), loop connecting αC-helix to IV (b.l.); gatekeeper (GK); loop connecting the hinge to the αD-helix (linker). With this background, we are ready to define the two interfaces.

Since the primary objective of this work is to determine the molecular determinants responsible for the molecular recognition of all PKI drugs, we focused our attention on finding common residues used by multiple drugs. The word “common” here means that an identical amino acid occupies the same location in the aligned complex structures. To do that, interacting residues for all 34 aligned complex structures were simultaneously displayed by the VMD program. If identical amino acids from multiple protein kinases overlap in space, a common residue is identified. The common residues for the back interface include V(11), A(15), K(17), E(24),V(36),T(45), and D(81). The front interface contains common residues L(3), F(47), G(51), L(77) and F(82).

Non-bonded interaction modes, including hydrogen bonding, salt bridge interactions, cation-π interaction, π-π stacking interactions, CH-π and XH-π (XH=NH, OH, SH) interactions, were systematically identified and tabulated. Subsequently, the strengths of intermolecular interaction energies between each drug and its surrounding protein residues were calculated at the RIJK RI-B2PLYP level in a pair-wise manner. For all the calculations considered in this study, the atomic coordinates of non-hydrogen atoms in PKI drugs and their interacting protein residues were extracted from the X-ray crystal structures of the complexes. The missing hydrogen atoms were patched to satisfy valence and their positions were determined by ab initio geometry optimization at the HF/6-31+G* level. During the geometry optimization processes, the positions of heavy (non-hydrogen) atoms were fixed. 

Based on the modes of non-bonded interactions identified above and their calculated interaction strengths, the molecular recognition of PKI drugs in various regions of the ATP-binding pockets was systematically analyzed, including the hinge region, the back interface, and the front interface.

### 2.1. Hydrogen Bonding in the Hinge Region 

As mentioned in the Introduction section, type I and II PKIs compete with ATP by mimicking the structure of ATP in the binding pocket. Because of a high abundance of backbone nitrogen and oxygen atoms in the hinge region, there exists an ample capacity for the formation of hydrogen bonds with PKI drugs. It is worth noting that the adenine base of ATP has the capacity to form five hydrogen bonds (two hydrogen bond donors at the N6 site and three hydrogen bond acceptors at the N1, N3, and N7 sites) with proteins. Indeed, a previous study has shown that hydrogen bonds between the adenine base and the protein backbones play an important role in the molecular recognition of ATP-binding proteins [27]. As competitive inhibitors of ATP binding site, PKI drugs have an energetic need to also form hydrogen bonds in the same pockets. Here, we aim at developing an understanding of the molecular recognition of FDA-approved kinase drugs with their surrounding residues inside the hinge region of protein kinases.

**Figure 2:**
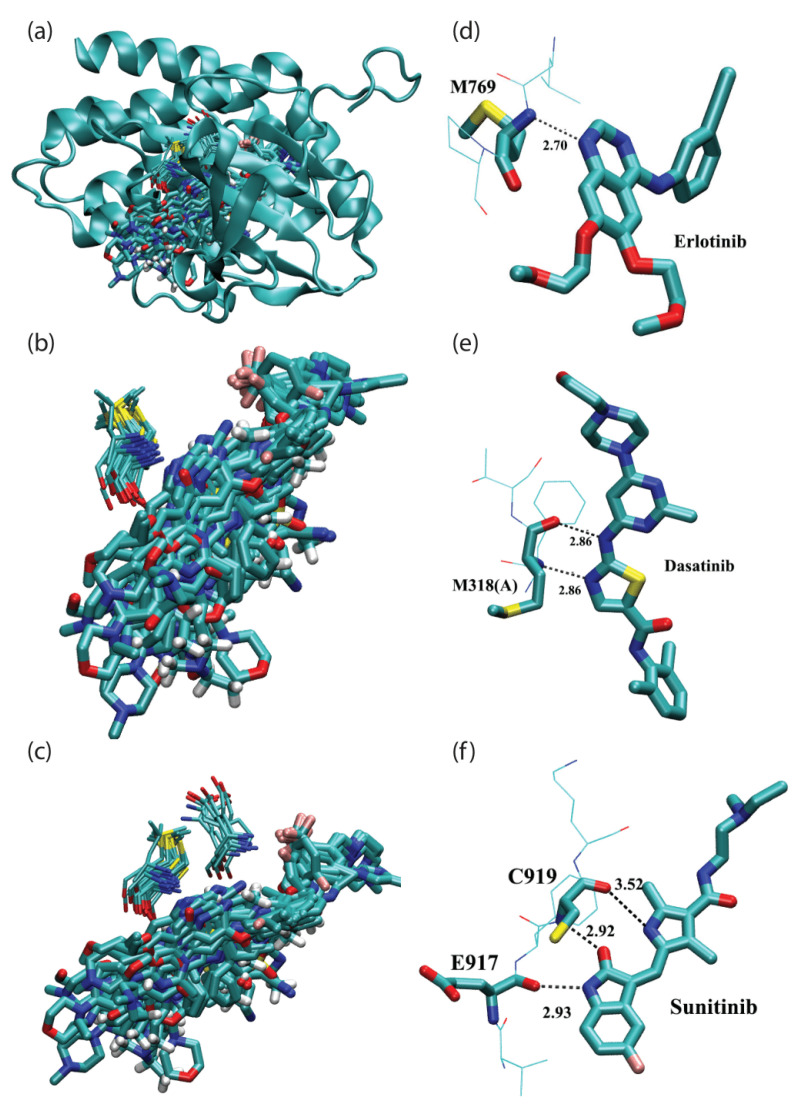
Hydrogen bonding of FDA-approved kinase drugs in the hinge region of protein kinases. (**a**) Overview of FDA-approved kinase drugs in their binding pockets inside protein kinases based on aligned drug-kinase complexes. Only one of the aligned protein kinases (PDB ID 1IEP) is shown in the cartoon representation. (**b**) Detailed configuration of FDA-approved kinase drugs (in thick licorice representation) that form hydrogen bonds with the main chain of a single residue (in thin licorice representation). (**c**) Detailed configuration of FDA-approved kinase drugs (in thick licorice representation) that form hydrogen bonds with two non-adjacent residues (in thin licorice representation). (**d**) A representative case of (**b**), displaying a single hydrogen bond based on the 2.6 Å crystal structure of the drug erlotinib bound with epidermal growth factor receptor (PDB ID: 1M17). (**e**) Another representative case of (**b**), displaying dual hydrogen bonds based on the 2.4 Å crystal structure of the drug dasatinib bound with proto-oncogene tyrosine-protein kinase ABL1 (PDB ID: 2GQG). (**f**) A representative case of (**c**), displaying triple hydrogen bonds based on the 2.8 Å crystal structure of the sunitinib bound with vascular endothelial growth factor receptor 2 (PDB ID: 4AGD).

Figure 2 depicts the hydrogen bonding of FDA-approved kinase drugs in the hinge region of protein kinases. Figure 2a shows an overlay of PKI drugs based on the aligned structures of all 34 protein kinases. For clarity, only one of the aligned protein kinases, proto-oncogene tyrosine-protein kinase ABL (PDB ID 1IEP), is shown as a reference. As displayed in Figure 2b, multiple PKI drugs form hydrogen bonds with the main chain of a single residue whose consensus sequence ID is 48. The remaining PKI drugs form hydrogen bonds with two non-adjacent residues (residue 48 and 46), which are shown in Figure 2c. For a more detailed look, representative cases for PKI drugs that form mono, dual and triple hydrogen bonds with the backbone N and O atoms of protein kinases are illustrated in Figure 2d–f, respectively. 

A complete list of hydrogen bonds between PKI drugs and the hinge region of protein kinases is given in Table 3. The second column of the table is the 3-letter ligand ID whose corresponding PKI drug name is tabulated in Table 1. The third and fourth columns list the H-bond donor (NH: the main chain amide nitrogen) and acceptor (O: the main chain O atom) from residue 48. The fifth column lists the hydrogen bond acceptor from a second non-adjacent residue whose consensus residue ID is 46. It can be seen from Table 3 that all PKI drugs studied here are capable of forming hydrogen bonds in the hinge region. Another intriguing finding is that all hydrogen bonds in the hinge region are formed between the protein backbone and FDA-approved kinase drugs. According to the number of hydrogen bonds, we can classify PKI drugs into three classes: mono, dual and triple hydrogen bond groups. There are 16 cases of mono hydrogen bonds formed between the main chain amide N atom and a PKI drug. Dual hydrogen bond occurred 17 times. Interestingly, the dual hydrogen bond can be formed with the main chain(s) of a single residue or two non-adjacent residues in the hinge region. It is worth noting that these patterns of dual hydrogen bonds are very similar to what was observed for hydrogen bonding of the adenine base with the hinge region in ATP-binding proteins [27]. There is even a case of triple hydrogen bonds, which occurs in the sunitinib bound with vascular endothelial growth factor receptor 2 (PDB ID: 4AGD). Representative cases for the three classes are displayed in Figure 2d–f, respectively. On average, PKI drugs form 1.6 hydrogen bonds with the backbone N and O atoms in the hinge region.

### 2.2. Intermolecular Interactions with Residues in the Back Interface

The role of the back interface in the molecular recognition of PKI drugs is studied in this section. As described early, visual examination of the 34 aligned drug-protein complexes revealed that seven “common” residues V(11), A(15), K(17), E(24),V(36),T(45), and D(81) on the back interface are positioned in the immediate surroundings of PKI drugs. Here, the number in parentheses represents the generic kinase sequence ID number according to the KLIFS numbering convention [26]. To facilitate later discussion, it is useful to introduce the identities of the “common” residues as listed in the second row of Table 4, using the KLIFS nomenclature of key binding motives [26]. T(45) was dubbed the gate keeper residue [24]. K(17) and E(24) are the two highly conserved residues crucial for the catalytic function of the kinase [82,83]. D(81) is part of the important DFG motif; the latter features catalytic aspartate that coordinates the magnesium binding [21,22]. 

**Figure 3:**
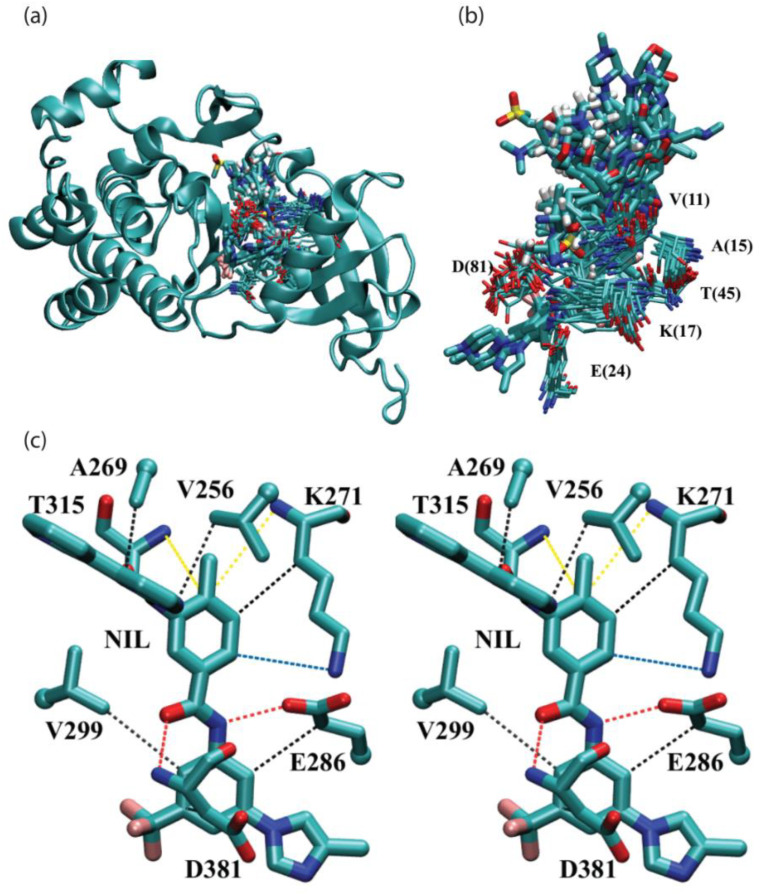
Intermolecular interactions between FDA-approved kinase drugs and “common” residues in the back interface of protein kinases. (**a**) Overview of FDA-approved kinase drugs surrounded by conserved residues in the back interface based on aligned drug-kinase complexes. Only one of the aligned protein kinases (PDB ID 1IEP) is shown in the cartoon representation. (**b**) Detailed view of FDA-approved kinase drugs surrounded by “common” residues V(11), A(15), K(17), E(24), V(36) (not visible, hidden behind), T(45), and D(81) on the back interface. (**c**) Stereo diagram of a representative case for (**b**); PKI drug nilotinib (NIL) interacts with residues Val256, Ala269, Lys271, Glu286, Val299, Thr315 and Asp381 on the back interface of proto-oncogene tyrosine-protein kinase ABL1 based on the 2.2 Å resolution crystal structure (PDB ID: 3CS9).

Figure 3a shows an overview of FDA-approved kinase drugs surrounded by “common” residues on the back interface. Figure 3b provides a more detailed view of residues V(11), A(15), K(17), E(24),V(36),T(45), and D(81) on the back interface that surrounds PKI drugs. Due to the limitation of 2D representation, the second valine residue V(36) is hidden behind. For the purpose of illustration, Figure 3c presents a stereo diagram of a PKI drug nilotinib interacting with residues Val256, Ala269, Lys271, Glu286, Val299, Thr315, and Asp381 on the back interface of proto-oncogene tyrosine-protein kinase ABL1 based on the 2.2 Å resolution crystal structure (PDB ID: 3CS9). In this case, molecular recognition of nilotinib in ABL1 is achieved by CH-π interaction, cation-π interaction, NH-π interactions, and H-bonding. Moreover, it offers a close-up view of the spatial distribution of, plus relative sequence IDs of, the seven surrounding residues.

A complete list of pair-wise intermolecular interaction energies between PKI drugs and surrounding residues on the back interface of protein kinases is presented in Table 4. Modes of intermolecular interactions between a given residue and PKI drugs are tabulated on the last row of Table 4. The numerical values represent the solution phase interaction energies ($∆E_{int\;}^{aq}\;)$ calculated according to Equation (2) of the Methods section. A negative value of the interaction energy indicates attractive interactions that stabilize binding. It can be seen from Table 4 that the vast majority of residues have the potential to generate favorable intermolecular interactions with PKI drugs. A thorough analysis of modes of intermolecular interactions showed that the highly conserved K(17) residue can form cation-π interactions, CH-π interactions, and NH-π interactions with aromatic moieties of PKI drugs. In 21 out of 34 drug-protein complexes, does the K(17) residue form favorable interactions with drugs, with an average interaction energy of –4.0 kcal/mol. Residues E(24) and D(81) can form favorable non-bonded interactions such as hydrogen bonds, CH-π interactions, and NH-π interactions to strengthen the binding of drugs with target proteins. In 7 out of 34 complexes, does the E(24) residue form favorable interactions, with an average energy of –2.7 kcal/mol. The same is true for the D(81) residue, occurring in 11 out of 34 complexes with an average energy of –1.0 kcal/mol.

The most important finding on the back interface is that there are a large number of π moiety involved interactions that stabilize PKI drug binding. From Table 4 and Figure 3, it is evident that V(11) and A(15) residues are highly conserved in this region; they can form lots of CH-π interactions with the aromatic moieties of PKI drugs. In 27 out of 34 drug-protein complexes, the A(15) residue forms favorable CH-π interactions with its interacting drugs, with an average interaction energy of −1.1 kcal/mol. The valine residue V(11) is highly conserved and presents in every complex to form favorable CH-π interactions with an average interaction energy of −1.7 kcal/mol; while for the other valine residue V(36), CH-π interactions with drugs occur in 15 out of 34 complexes, with an average interaction energy of –1.4 kcal/mol. Besides, in 11 out of 34 complexes, does the gate keeper residue T(45) forms favorable interactions (H-bonding, CH-π interactions, NH-π interactions, OH-π interactions) with drugs, yielding an average interaction energy of −1.5 kcal/mol.

From the quantitative analysis presented above, key insights into the molecular recognition of PKI drugs emerge. On the back interface of the aligned proteins, there are five highly conserved residues (V(11), A(15), K(17), T(45) and D(81)) that are involved in the molecular recognition of FDA-approved kinase drugs via multiple modes of intermolecular interactions including CH-π interactions, hydrogen bonding, cation-π interaction, NH-π interaction, and OH-π interaction. 

### 2.3. Intermolecular Interactions with Residues on the Front Interface

**Figure 4:**
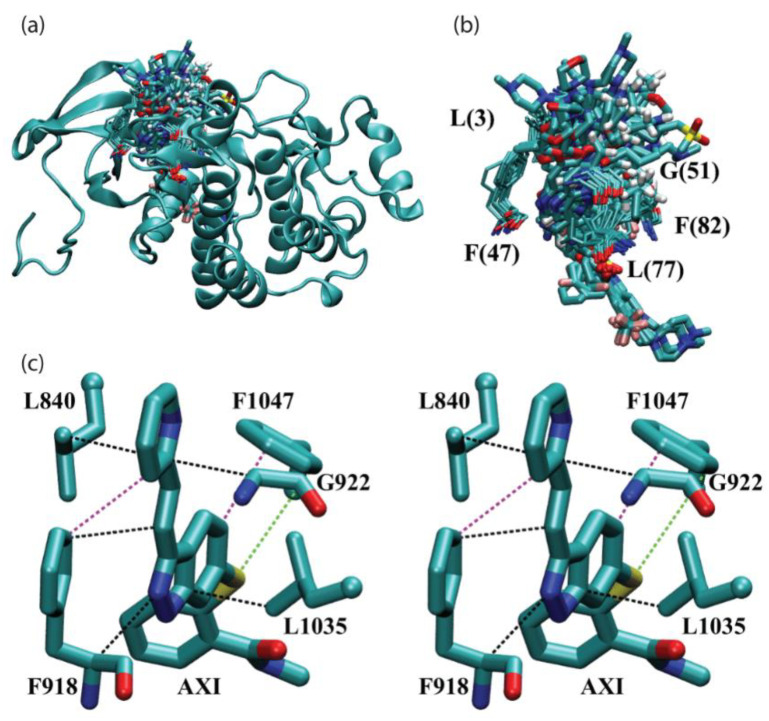
Intermolecular interactions between FDA-approved kinase drugs and “common” residues on the front interface of protein kinases. (**a**) Overview of FDA-approved kinase drugs surrounded by hydrophobic residues in the front interface based on aligned drug-kinase complexes. Only one of the aligned protein kinases (PDB ID 1IEP) is shown in the cartoon representation. (**b**) Detailed view of FDA-approved kinase drugs surrounded by hydrophobic residues L(3), F(47), G(51), L(77) and F(82) (partially visible, hidden behind) on the front interface. (**c**) Stereo diagram of a representative case for (**b**); PKI drug axitinib (AXI) interacting with hydrophobic residues Leu840, Phe918, GLY922, Leu1035 and Phe1047 on the front interface of vascular endothelial growth factor receptor 2 (PDB ID: 4AG8).

The role of the front interface in the molecular recognition of PKI drugs is investigated in this section. As stated early, based on the aligned drug-protein complexes, it was found that five hydrophobic residues L(3), F(47), G(51), L(77) and F(82) on the front interface are positioned in the immediate surroundings of PKI drugs. Figure 4a presents an overview of FDA-approved kinase drugs surrounded by those hydrophobic residues on the front interface. Figure 4b provides a more detailed view of residues L(3), F(47), G(51), L(77) and F(82) on the front interface that surround PKI drugs. Due to the limitation of 2D representation, the second phenylalanine residue F(82) is hidden behind. For the purpose of illustration, Figure 4c shows a stereo diagram of a representative PKI drug axitinib (AXI) interacting with hydrophobic residues Leu840, Phe918, GLY922, Leu1035, and Phe1047 on the front interface of vascular endothelial growth factor receptor 2 (PDB ID: 4AG8). As depicted in the figure, molecular recognition of axitinib in VEGFR2 is achieved by CH-π interaction, π-π stacking interactions, and SH-π interaction. Moreover, it offers a close-up view of the spatial distribution of, plus relative sequence IDs of, the five surrounding residues.

A complete list of pair-wise intermolecular interaction energies between PKI drugs and surrounding residues on the front interface of protein kinases is presented in Table 5. The numerical values represent the solution phase interaction energies ($∆E_{int\;}^{aq}\;)$ calculated according to Equation (2) of the Methods section. As done in the case of the back interface, it is useful to introduce the identities of the “common” residues as listed in the second row of Table 5, using the KLIFS nomenclature of key binding motives [26]. Notable identities of “common” residues on the front interface are described below. F(47) is located on the hinge. G(51) sits on the linker that connects the hinge to the αD-helix. F(82) is part of the DFG motif. 

The most striking observation on the front interface is the abundance π moiety involved interactions that stabilize PKI drug binding. The last row of Table 5 lists the modes of intermolecular interactions between a given residue and PKI drugs. From both Table 5 and Figure 4, it is evident that both Leu and Phe residues are highly conserved in this region; they can form lots of CH-π interactions and π -π stacking interactions with PKI drugs. Altogether, the two conserved Leu residues form a total of 52 favorable CH-π interactions with PKI drugs, with an average energy of –2.1 kcal/mol. The two Phe residues are involved in CH-π and π -π stacking interactions on 21 occasions. In this case, the average strengths of −2.2 kcal/mol for Phe residues intermolecular interactions are significantly larger. As shown in Table 5, in 22 of the 34 complexes, the GLY residue forms favorable interactions with PKI drugs, with an average value of –1.5 kcal/mol.

The above quantitative analysis leads to key insights into the molecular recognition of PKI drugs in the hydrophobic region. First, the front interface is occupied by five hydrophobic residues L(3), F(47), G(51), L(77) and F(82). By virtue of their capacity to form multiple CH-π and π -π stacking interactions with aromatic moieties of PKI drugs, they make a sizeable energetic contribution to the binding of PKI drugs. Second, it is worth noting that the conserved Leu and Phe residues used by PKI drugs for binding (mainly via CH-π and π -π stacking interactions) are not used by ATP, which provides all ATP-competitive PKI drugs that possess aromatic functional groups an energetically competitive advantage against ATP. 

### 2.4. Illustrative Example of Molecular Recognition of PKI Drugs in Protein Kinases

To gain a better understanding of the entire binding pockets of FDA-approved kinase drugs in their targeted protein kinases, molecular recognition in one representative example of drug-protein complexes was systematically studied. For this purpose, the complex between the PKI drug lenvatinib and its target vascular endothelial growth factor receptor 2 (VEGFR2) is presented. Our goal here is to assess the strengths and the relative importance of different modes of intermolecular interactions in drug binding, as well as the relative contribution of all non-covalent interactions originated from different regions of the ATP binding pockets.

**Figure 5:**
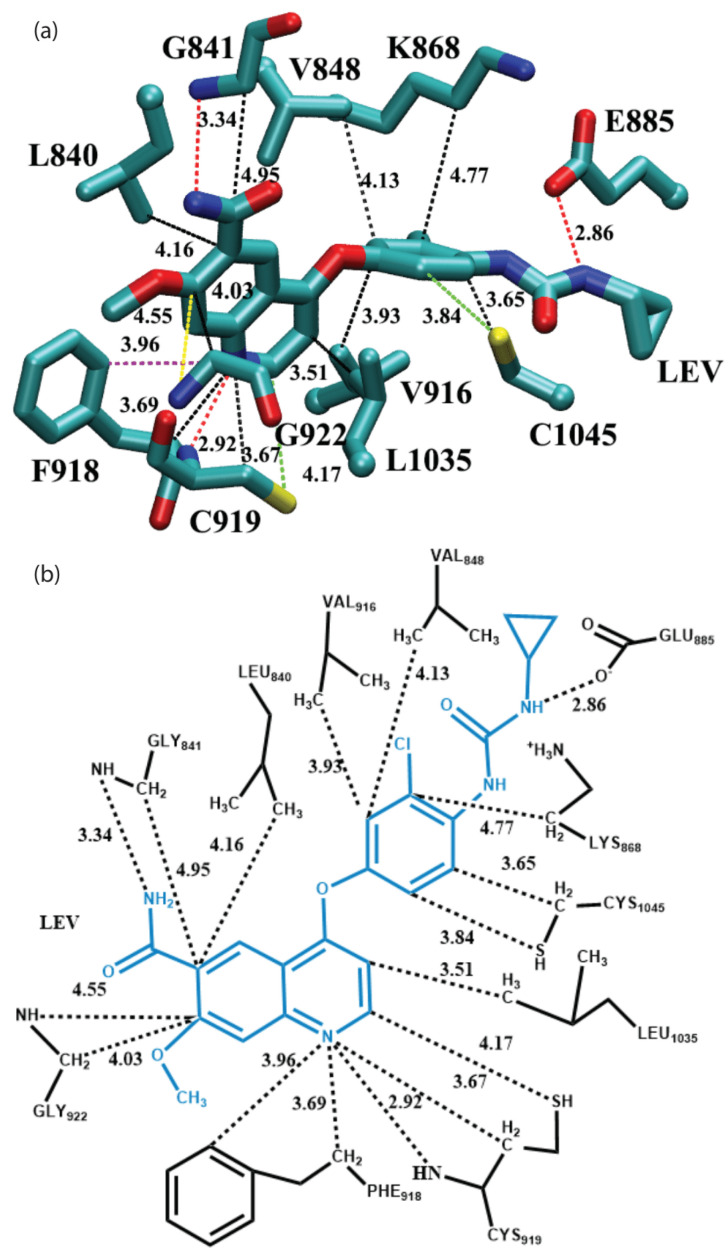
Modes of intermolecular interactions between lenvatinib and residues from vascular endothelial growth factor receptor 2 (PDB ID: 3WZD). (**a**) three dimensional arrangement of lenvatinib and its interacting residues that are within 5.5 Å of lenvatinib based on the 1.57 Å resolution X-ray crystal structure (PDB ID: 3WZD). Dash lines indicate interatomic distance in Å for various intermolecular interactions (color code: hydrogen bonding in red, π-π stacking interactions in purple, CH-π interactions in black, NH-π interactions in yellow, and SH-π interactions in green) (**b**). A schematic intermolecular interaction map between lenvatinib and its interacting residues, with dashed lines indicating the interatomic distance in Å.

Modes of non-bonded interactions, such as hydrogen bond, salt bridge, π-π stacking interactions, cation-π interactions, CH-π interactions, and XH-π interactions (XH=NH, OH, SH) were systematically identified in the lenvatinib binding pocket of the vascular endothelial growth factor receptor 2 (VEGFR2). Subsequently, strengths of intermolecular interaction for each mode were quantified by means of the supramolecular approach at the RIJK RI-B2PLYP level followed by solvation correction. The detailed interaction patterns are displayed in Figure 5. Figure 5a shows the three-dimensional arrangement of lenvatinib and its interacting residues that are within 5.5 Å of lenvatinib based on the 1.57 Å resolution X-ray crystal structure of lenvatinib bound VEGFR 2 (PDB ID: 3WZD [69]). Figure 5b displays a schematic intermolecular interaction map between lenvatinib and its interacting residues. Lenvatinib interacts with its target protein VEGFR2 via hydrogen bonding, π-π stacking interactions, CH-π, NH-π, and SH-π interactions. 

Pairwise intermolecular interaction energies between lenvatinib and residues from vascular endothelial growth factor receptor 2 are listed in Table 6 where $∆E_{B2\mathrm{PLYP}\;}^{g}$ represents the gas phase intermolecular interaction energy calculated at the B2PLYP level. The dehydration energy $∆E_{Deh}^{}$ is defined in Equation (3) of the Methods section. The solution phase interaction energies $∆E_{int\;}^{aq}$ are calculated according to Equation (2) of the Methods section. The modes of intermolecular interactions for a given residue are listed in the third column. Some residues can form multiple modes of interactions with lenvatinib.

In the hinge region, the backbone amide N atom of Cys919 forms a hydrogen bond with lenvatinib, with a distance of 2.92 Å. Interestingly, Cys919 is also interacting with lenvatinib by CH-π, and SH-π interactions, in addition to hydrogen-bonding. Altogether, the multimode intermolecular interactions produce a solution phase interaction energy of −2.2 kcal/mol. There are numerous CH-π interactions between lenvatinib and its surrounding residues. On the back interface, Val848, Lys868 and Val916 can form CH-π interactions with lenvatinib, while on the front interface, Leu840, Gly922, Leu1035 and can also form CH-π interaction, NH-π interaction, and π-π stacking interactions with the drug. The strengths of all CH-π interactions combined dominate the binding energy between the drug and protein. 

In addition to Cys919, four other residues Gly841, Phe918, Gly922, and Cys1045 can also form multimode intermolecular interactions. Phe918 participates in CH-π and π-π stacking interactions with the drug. Cys1045 can form both SH-π and CH-π interactions with lenvatinib. As shown in Table 6, the multimode intermolecular interactions resulted in enhanced strengths of interaction energies. In addition to the hydrogen bond in the hinge region, lenvatinib also forms hydrogen bonds with Gly841 and Glu885, with a distance of 3.34 Å and 2.86 Å, respectively. The latter forms between the side chain of Glu885 and the drug. 

In summary, molecular recognition of lenvatinib in its target vascular endothelial growth factor receptor 2 is achieved by a combination of multiple modes of intermolecular interactions: Hydrogen bonding in the hinge region and outside; CH-π interaction, and π-π stacking interactions on both the back interface and front interface; plus the NH-π interaction originating from the backbone amide nitrogen atom.

## 3. Discussion

How do FDA-approved kinase inhibitor drugs compete with ATP in its own binding pocket? This is the question we attempted to address in this work. To do that, we determined the molecular determinants responsible for the molecular recognition of FDA-approved PKI drugs in their targeted kinases. 

As was the case in similar types of studies, we started with the identification of non-bonded interactions between each PKI drug and residues in its binding pocket of the targeted protein. Subsequently, the strengths of non-bonded interactions were calculated by means of an advanced level double hybrid DFT method B2PLYP [84]. The latter was identified in a benchmark study by us as the best-performing DFT method for the calculation of non-bonded interactions in terms of both accuracy and computational efficiency in comparison with the highly accurate CCSD(T) method [85]. It is well known that the strengths of non-bonded interactions depend strongly on both the distance and orientation of interacting molecular species. Therefore, accurate quantification of interaction strengths for non-bonded interaction energies provided us with a solid foundation to deal with the task at hand. 

Based on modes of non-bonded interactions and their calculated interaction strengths, the molecular recognition of PKI drugs in the ATP-binding pockets was systematically analyzed. This resulted in the following findings:

(1) Mimicking the adenine base of ATP, all FDA-approved PKI drugs studied here form hydrogen bonds with the backbone N and O atoms in the hinge region. The number of hydrogen bonds formed varied from one to three. Interestingly, patterns of hydrogen bonds are very similar to what was observed for hydrogen bonding of the adenine base with the hinge region in ATP-binding proteins [27]. It is worth noting that the formation of hydrogen bond(s) with the backbone of the hinge gives PKI drugs the added binding affinity and the much-needed directionality, but selectivity is sacrificed due to the sole use of the main chain only;
(2) Aromatic rings of FDA-approved drugs reach far and deep into the hydrophobic regions I and II, forming multiple non-bonded π-interactions with hydrophobic residues, but ATP itself does not utilize these regions extensively [25]. As can be seen from Table 1, all the FDA-approved PKI drugs studied here feature two or more aromatic rings. In comparison to the adenine base, FDA Approved PKI drugs have a much deeper reach into the hydrophobic regions I and II to form a multitude of non-bonded π-interactions, particularly CH-π interactions with aliphatic residues L(3), V(11), A(15), V(36), G(51), L(77) and π-π stacking interactions with aromatic residues F(47) and F(82). As illustrated in Section 2.4, quantitatively, CH-π interactions make a dominant contribution to binding energy for molecular recognition of lenvatinib in its target vascular endothelial growth factor receptor 2. As noted early, ATP is not able to form extensive non-bonded interactions with hydrophobic residues in hydrophobic regions I and II. Thus, FDA-approved PKI drugs win the competition against ATP by their constituting aromatic rings. That explains why all the FDA-approved drugs are aromatic rich. It is worth noting that a previous study of more than two thousand protein kinase inhibitors reached a similar conclusion that aromatic rings play an important role in the molecular recognition of protein kinase inhibitors [28].
(3) Based on all 34 aligned drug-protein complex structures, we identified residues that are used by multiple PKI drugs for binding interaction, and named them “common residues”. Bear in mind that the words “common” here means an identical amino acid occupies the same location in the aligned complex structures. It was found that a total of 12 “common” residues in the binding pockets of protein kinases participate in the molecular recognition of FDA-approved PKI drugs. As described in the Results section, the “common” residues on both the back interface and the front interface include L(3), V(11), A(15), K(17), E(24), V(36), T(45), F(47), G(51), L(77), D(81) and F(82). Before discussing the significance of this finding, we want to call readers’ attention to the identities of these “common” residues. K(17) and E(24) are the two highly conserved residues crucial for the catalytic function of the kinase [82,83]. D(81) and F(82) belong to the important DFG motif; the latter features catalytic aspartate that coordinates the magnesium binding [21,22]. T(45) was dubbed the gate keeper residue [24]. F(47) is located on the hinge region and G(51) sits on the linker that connects the hinge to the αD-helix. For either structural or functional reasons, K(17), E(24), D(81) and D(82) are highly conserved thorough out the human kinome; V(11), A(15), and L(77) are nearly conserved; L(3) and V(36) are somewhat conserved, but to a much less extent [26,86]. As shown in Table 4 and Table 5, L(3), V(11), A(15), K(17), G(51), L(77), and D(81) were major targets of non-bonded interactions from FDA-approved drugs, occurring with very high frequency (i.e., very “common”). Targeting highly conserved residues with intrinsically important structural and biological functional roles allows the FDA-approved drugs to block protein kinases from their enzymatic function of phosphotransfer, and to gain higher binding affinity at the same time. This binding affinity gain for the FDA-approved drugs is particularly important when competing against ATP which has a very high cellular concentration. However, targeting highly conserved residues in protein kinases can lead to promiscuous PKI drugs [87]. Although the formation of hydrogen bond(s) with the backbone of the hinge gives PKI drugs the added binding affinity and the much-needed directionality, there is no potential to gain selectivity. That may be why so many FDA-approved PKI drugs are known to have multiple targets, as listed in Table S1. For example, imatinib was originally designed as an inhibitor of the BCR-ABL kinase as a treatment for chronic myeloid leukemia (CML). It can also inhibit the C-KIT and PDGFR-alpha, in addition to BCR-ABL. Moreover, off-target-mediated toxicity caused by a lack of selectivity was one of the major challenges facing the PKI drug discovery community. This work suggests a road map for future PKI drug design, i.e., targeting non-conserved residues in the ATP binding pocket to gain better selectivity so as to avoid off-target-mediated toxicity.

Ninety percent of clinical drug development fails [88], with drug toxicity originated from a lack of selectivity as one of the direct causes [89]. So the selectivity profiles of FDA approved PKI drugs represent an important matter that merits further discussion. For the purpose, difference in molecular recognition of PKI drugs by the two major types of protein kinases are analyzed here. As shown in Table 4 and Table 5, 27 tyrosine kinases and 7 serine/threonine kinases are tabulated in groups separated by a dividing line. The last row of Table 4 and Table 5 lists the modes of intermolecular interactions between a given residue and PKI drugs. A major difference in molecular recognition of PKI drugs between tyrosine kinases and serine/threonine kinases occurred in the front interface. In the case of tyrosine kinases, residues L(3), F(47), G(51) and L(77) are highly conserved, and form strong favorable interactions with FDA-approved PKI drugs via CH-π interactions, π-π stacking interactions, NH-π interactions, cation-π interactions, and hydrogen bonding. In contrast, serine/threonine kinases do not feature L(3), F(47), G(51) residues that can interact with FDA-approved PKI drugs. This distinction between interacting residues of tyrosine kinases and those of serine/threonine kinases can be advantageous to drug binding specificity, which improves drug selectivity.

From the perspective of drug design, an important lesson can be learned from FDA-approved PKI drugs, namely, the energetic benefit of aromatic functional groups in drug molecules. Although charged and polar functional groups possess the capacity of forming strong non-bonded interactions such as salt bridge and hydrogen bonding in the gas phase, the strengths of these electrostatic types of interactions attenuate substantially in the solution phase. That is understandable since both the drug and protein are solvated before binding together; they both lose part of their solvation shell upon binding, which incurs a heavy dehydration energy cost. The dehydration energy cost is particularly pronounced in the case of charged and polar groups. Due to its electrostatic nature, cation-π interaction also has a large dehydration energy cost. So the net energy gain for the solution phase non-bonded interactions like hydrogen bonding, salt bridge interaction and cation-π interaction is not as large as it appears in the gas phase. In contrast, aromatic and nonpolar functional groups dislike water to start with. After all, we have a saying “like dissolves like” in chemistry. The protein interior is generally non-polar, with a high content of non-polar and aromatic residues. Therefore, non-bonded interactions dominated by dispersion forces, including CH-π interactions and π-π stacking interactions, do not suffer from the high cost of dehydration energy (see Table 6). Although the gas phase interaction energies for CH-π interactions and π-π stacking interactions are not very large, there is virtually no energetic cost for dehydration. In the end, the net energetic gain for the solution phase non-bonded interactions like CH-π interactions and π-π stacking interactions remains. Finally, it should be noted that CH-π interactions with the aromatic rings of drugs are not limited to aliphatic residues Ala, Leu, Ile, and Val. The middle methylene groups of Lys, Thr, and Cys residues also have the potential to form multiple CH-π interactions with drugs. For example, as shown in Table 6, the middle methylene groups of Lys868 of vascular endothelial growth factor receptor 2 formed CH-π interactions (−1.1 kcal/mol) with Lenvatinib. The residue Cys1045 also participated in CH-π interactions with Lenvatinib. As a general principle, from the point of view of binding energetics, the importance of CH-π interactions and π-π stacking interactions in molecular recognition must not be underestimated.

From the perspective of theoretical treatment, there is a pitfall associated with using the X-ray crystal structures directly for calculations of non-bonded interaction energies. Due to the technical limitations of X-ray crystal structure determination, there is an intrinsic uncertainty in the atomic coordinates in the reported PDB structures [90]. The coordinate error may in turn lead to errors in calculated non-bonded interaction energies. This problem is particularly pronounced in the case of hydrogen bonding between T315 and the PKI drug Dasatinib based on a 2.40 Å resolution X-ray crystal of the drug-protein complex [63]. The calculated hydrogen bonding energy is highly repulsive (+9.7 kcal/mol), as listed in Table 4. A careful examination of the coordinates revealed serious steric clashes in geometry, which led to this unphysical interaction energy for the hydrogen bond. In principle, the steric clash caused by this coordinate error can be alleviated by performing a full geometry optimization of the drug-protein complex. However, given the large number of complexes studied in this work, it is computationally not feasible to do so. 

In conclusion, our findings led to the establishment of the following pharmacophore model for FDA-approved PKI drugs: a small molecule features a scaffold of one or more aromatic rings that are linked with multiple hydrophilic functional groups. The former play the structural role of acting as a scaffold and the functional role of participating in CH-π interactions, π-π stacking interactions, cation-π interactions with residues in hydrophobic regions I, II, and the adenine region. The latter ensure water solubility and form hydrogen bonds with the hinge region and other hydrophilic residues of the ATP binding pocket. It is our expectation that this pharmacophore model will have the promise to profoundly impact lead optimization in future structure-based and ligand-based designs of potent and selective ATP binding site targeted PKI drugs. For the readers of this Special Issue on “New Advances in the Development of Kinase Inhibitors”, the gained insights on the molecular determinants responsible for molecular recognition of FDA-approved PKI drugs in their targeted kinases will be especially valuable. 

## 4. Theory and Methods

### 4.1. Data Mining and 3D Alignment of Structures

A large-scale data mining of the PDB was carried out to establish a database of PKI drugs bound to their respective proteins. Only high-resolution (3.5 Å or better) X-ray crystal structures of protein kinases complexed with bound PKI drugs were retained for further analysis. In the case of redundant entries (i.e., the same drug bound to the same protein kinase), the entry with the higher resolution was kept. 

The resulting drug-protein complexes were superimposed by means of pairwise 3D structure alignment based on a single reference structure (PDB ID 1IEP). For this purpose, the FATCAT algorithm as implemented on the RCSB PDB site was utilized for this rigid body alignment [49,50]. 

### 4.2. Quantum Chemical Calculation of Intermolecular Interaction Energies

The binding energy for the ligand (A) protein (B) complex formation in the solution can be evaluated indirectly by means of the following thermodynamics cycle.

$$A(\mathrm{aq})+B(\mathrm{aq})\;\overset{∆E_{int\;}^{aq}}{\to }\;\mathrm{AB}\left(\mathrm{aq}\right)$$

(1) $$∆G_{A}^{sol}↑\;\;∆G_{B}^{sol}↑\;↑∆G_{AB}^{sol}$$

$$A(g)+B(g)\;\underset{∆E_{int\;}^{g}}{\to }\;\mathrm{AB}\left(g\right)$$

Only salient features will be highlighted here. For a detailed description of the scheme, interested readers can refer to Reference [28]. According to the scheme, the binding energy for complex formation in the solution can be evaluated indirectly by calculating intermolecular interaction energies in the gas phase, $∆E_{int\;}^{g}$, followed by a correction for the dehydration energy $∆E_{Deh}^{}$:

(2) $$∆E_{int\;}^{aq}=∆E_{int\;}^{g}+∆E_{Deh}^{}\;$$

The gas phase intermolecular interaction energies between a drug and its surrounding residues in protein kinases, $∆E_{int\;}^{g}$, were calculated using the supermolecular approach. The calculations were carried out using the ORCA 4.0 program [91] by means of the double-hybrid density functional method B2PLYP [84] with the def2-QZVP basis set [92]. Grimme’s D3BJ dispersion correction [93] was applied for a proper account of dispersion interactions. For efficiency, B2PLYP was implemented with the resolution of identity (RI) approximation for the perturbation step and RIJK [94] for the SCF step. The basis set superposition error (BSSE) was corrected by the Boys and Bernardi Counter Poise Method [95].

The dehydration energy for the complex formation is defined (see Equation (1)) by

(3) $$∆E_{Deh\;}^{}=∆G_{AB}^{sol}-∆G_{A}^{sol}-∆G_{B}^{sol}$$

where $∆G_{i}^{sol}$, i = *AB*, *A*, *B* represents the free energies of solvation for the complex *AB*, and the monomers *A*, *B*, respectively. The SM5.42R Solvation Model of Cramer and Truhlar as implemented in the 2008 R1 version of GAMESS [96] was adopted for the evaluation of those free energies of solvation [97,98].

## Figures and Tables

**Table 1:** Molecular structures of 30 FDA-approved PKI drugs.

Name ^a^	Molecular Formula	Name ^a^	Molecular Formula
Abemaciclib (6ZV)	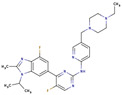	Imatinib (STI)	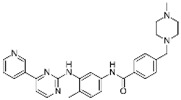
Afatinib (0WN)	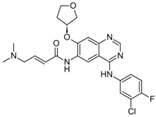	Lapatinib (FMM)	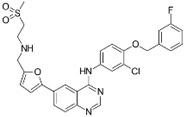
Alectinib (EMH)	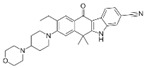	Lenvatinib (LEV)	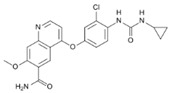
Axitinib (AXI)	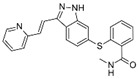	Neratinib (HKI)	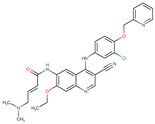
Bosutinib (DB8)	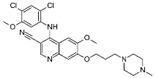	Nilotinib (NIL)	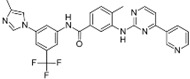
Brigatinib (6GY)	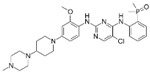	Nintedanib (XIN)	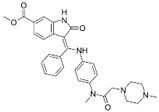
Ceritinib (4MK)	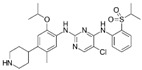	Palbociclib (LQQ)	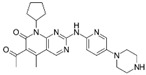
Cobimetinib (EUI)	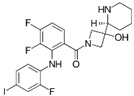	Ponatinib (0LI)	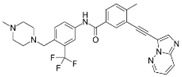
Crizotinib (VGH)	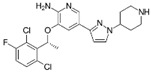	Ribociclib (6ZZ)	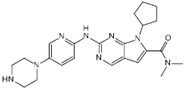
Dabrafenib (P06)	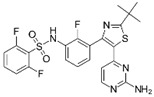	Ruxolitinib (RXT)	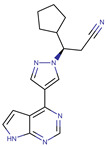
Dacomitinib (1C9)	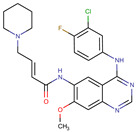	Sorafenib (BAX)	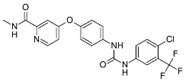
Dasatinib (1N1)	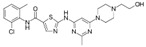	Sunitinib (B49)	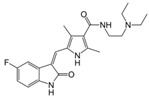
Erlotinib (AQ4)	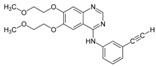	Tofacitinib (MI1)	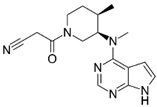
Gefitinib (IRE)	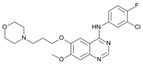	Vandetanib (ZD6)	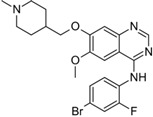
Ibrutinib (8E8)	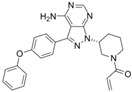	Vemurafenib (032)	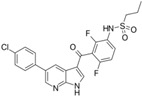

a: This column lists the name and ligand ID of FDA-approved kinase drugs.

**Table 2:** List of PKI drug-protein complexes.

Drug	Ligand ID	Protein Kinase Name	Type ^a^	PDB ID	Resolution (Å)	Ref.
Abemaciclib	6ZV	Cyclin-dependent kinase 6	STK	5L2S	2.27	[51]
Afatinib	0WN	Epidermal growth factor receptor	TK	4G5J	2.80	[52]
Alectinib	EMH	ALK tyrosine kinase receptor	TK	3AOX	1.75	[53]
Axitinib	AXI	Vascular endothelial growth factor receptor 2	TK	4AG8	1.95	[54]
Bosutinib	DB8	Tyrosine-protein kinase ABL1	TK	3UE4	2.42	[55]
Brigatinib	6GY	ALK tyrosine kinase receptor	TK	6MX8	1.96	[56]
Ceritinib	4MK	ALK tyrosine kinase receptor	TK	4MKC	2.01	[57]
Cobimetinib	EUI	Dual specificity mitogen-activated protein kinase 1	STK	4AN2	2.5	[58]
Crizotinib	VGH	Proto-oncogene tyrosine-protein kinase ros	TK	3ZBF	2.2	[59]
Crizotinib	VGH	Hepatocyte growth factor receptor	TK	2WGJ	2.0	[60]
Dabrafenib	P06	Serine/threonine-protein kinase B-RAF	STK	5CSW	2.66	[61]
Dacomitinib	1C9	Epidermal growth factor receptor	TK	4I23	2.8	[62]
Dasatinib	1N1	Proto-oncogene tyrosine-protein kinase ABL1	TK	2GQG	2.4	[63]
Erlotinib	AQ4	Epidermal growth factor receptor	TK	1M17	2.60	[64]
Gefitinib	IRE	Epidermal growth factor receptor	TK	4WKQ	1.85	[65]
Ibrutinib	8E8	Tyrosine-protein kinase BTK	TK	5P9J	1.08	[66]
Imatinib	STI	proto-oncogene tyrosine-protein kinase ABL	TK	1IEP	2.10	[67]
Lapatinib	FMM	Epidermal growth factor receptor	TK	1XKK	2.40	[68]
Lenvatinib	LEV	Vascular endothelial growth factor receptor 2	TK	3WZD	1.57	[69]
Neratinib	HKI	Epidermal growth factor receptor	TK	2JIV	3.50	[70]
Nilotinib	NIL	Proto-oncogene tyrosine-protein kinase ABL1	TK	3CS9	2.21	[71]
Nintedanib	XIN	Vascular endothelial growth factor receptor 2	TK	3C7Q	2.10	[72]
Palbociclib	LQQ	Cyclin-dependent kinase 6	STK	5L2I	2.75	[51]
Ponatinib	0LI	Mast/stem cell growth factor receptor Kit	TK	4U0I	2.00	[73]
Ponatinib	0LI	Tyrosine-protein kinase ABL1	TK	3OXZ	2.20	[74]
Ribociclib	6ZZ	Cyclin-dependent kinase 6	STK	5L2T	2.37	[51]
Ruxolitinib	RXT	Proto-oncogene tyrosine-protein kinase Src	TK	4U5J	2.26	[75]
Sorafenib	BAX	Vascular endothelial growth factor receptor 2	TK	4ASD	2.03	[54]
Sorafenib	BAX	B-RAF proto-oncogene serine/threonine kinase	STK	1UWH	2.95	[76]
Sunitinib	B49	Vascular endothelial growth factor receptor 2	TK	4AGD	2.81	[54]
Tofacitinib	MI1	Tyrosine-protein kinase JAK3	TK	3LXK	2.00	[77]
Tofacitinib	MI1	Tyrosine-protein kinase	TK	3EYG	1.90	[78]
Vandetanib	ZD6	RET Proto-oncogene tyrosine-protein kinase	TK	2IVU	2.50	[79]
Vemurafenib	032	AKAP9-BRAF fusion protein	STK	3OG7	2.45	[80]

a. Types of protein kinases: STK=serine/threonine kinases, TK=tyrosine kinases.

**Table 3:** List of H-bonds in the hinge region of the drug-protein complex.

PDB ID	Ligand	Residue 48 ^a^		Residue 46 ^a^
		HB Donor	HB Acceptor	HB Acceptor
1IEP	STI	M318(NH)	-	-
1M17	AQ4	M769(NH)	-	-
1UWH	BAX	C531(NH)	C531(O)	-
1XKK	FMM	M793(NH)	-	-
2GQG	1N1	M318(NH)	M318(O)	-
2IVU	ZD6	A807(NH)	-	-
2JIV	HKI	M793(NH)	-	-
2WGJ	VGH	M1160(NH)	-	P1158(O)
3AOX	EMH	M1199(NH)	-	-
3C7Q	XIN	C919(NH)	-	E917(O)
3CS9	NIL	M318(NH)	-	-
3EYG	MI1	L959(NH)	-	E957(O)
3LXK	MI1	L905(NH)	-	E903(O)
3OG7	032	C532(NH)	-	Q530(O)
3OXZ	0LI	M318(NH)	-	-
3UE4	DB8	M318(NH)	-	-
3WZD	LEV	C919(NH)	-	-
3ZBF	VGH	M2029(NH)	-	E2027(O)
4AG8	AXI	C919(NH)	-	-
4AGD	B49	C919(NH)	C919(O)	E917(O)
4AN2	EUI	S212(NH)	-	-
4ASD	BAX	C919(NH)	C919(O)	-
4G5J	0WN	M793(NH)	-	-
4I23	1C9	M793(NH)	-	-
4MKC	4MK	M1199(NH)	M1199(O)	-
4U0I	0LI	C673(NH)	-	-
4U5J	RXT	M341(NH)	M341(O)	-
4WKQ	IRE	M793(NH)	-	-
5CSW	P06	C532(NH)	C532(O)	-
5L2I	LQQ	V101(NH)	V101(O)	-
5L2S	6ZV	V101(NH)	V101(O)	-
5L2T	6ZZ	V101(NH)	V101(O)	-
5P9J	8E8	M477(NH)	-	E475(O)
6MX8	6GY	M1199(NH)	M1199(O)	-

a. Residue ID is numbered according to the KLIFS numbering convention [26].

**Table 4:** Pairwise interaction energies ($∆E_{int\;}^{aq}\;\mathrm{in}\;\mathrm{kcal}/\mathrm{mol}$ ) between PKI drugs and surrounding residues in the back interface ^a^.

PDB ID	Ligand ID	V(11) ^b^	A(15)	K(17)	E(24)	V(36)	T(45)	D(81)
Structure Elements		β Sheet II	β Sheet III	β Sheet III	αC-Helix	-	Gate Keeper	DFG Motif
1IEP	STI	−0.5	−0.8	- ^c^	0.3	−1.7	−2.1	0.3
1M17	AQ4	−2.4	−1.3	−5.6	-	-	−1.1	1.6
1XKK	FMM	−3.4	-	−6.6	-	-	−2.1	−1.5
2GQG	1N1	−0.9	−1.1	−3.7	−0.9	5.4	9.7	0.4
2IVU	ZD6	−4.0	−0.8	−6.1	-	-	-	−0.9
2JIV	HKI	−1.9	−1.3	−0.6	-	-	-	1.8
2WGJ	VGH	−2.5	−1.1	-	-	-	-	0.2
3AOX	EMH	0.5	1.2	0.9	-	1.6	-	-
3C7Q	XIN	−3.2	−1.9	−3.1	-	−0.8	-	-
3CS9	NIL	−1.4	−0.4	−3.9	−5.6	−1.2	0.8	0.0
3EYG	MI1	−1.3	−0.6	-	-	−0.9	-	-
3LXK	MI1	−1.3	−0.2	-	-	−0.9	-	-
3OXZ	0LI	−1.7	−0.8	−1.7	−1.7	−4.0	−2.2	3.3
3UE4	DB8	−4.8	−1.5	−6.6	-	−0.7	4.2	-
3WZD	LEV	−2.3	-	−1.1	0.4	−2.4	-	-
3ZBF	VGH	−0.4	−0.9	-	-	-	-	-
4AG8	AXI	−3.5	−1.7	−4.4	−0.9	−1.6	-	−0.9
4AGD	B49	0.0	0.5	2.5	-	0.8	-	−0.8
4ASD	BAX	−1.3	−1.8	−3.6	−2.7	−1.9	-	0.3
4G5J	0WN	−0.2	-	−4.2	-	-	0.4	−1.1
4I23	1C9	−2.4	−1.0	−0.7	-	-	−1.4	0.3
4MKC	4MK	−0.9	−1.0	-	-	-	-	-
4U0I	0LI	−1.6	−0.8	−4.5	−3.2	−2.6	−1.4	1.5
4U5J	RXT	−1.0	−0.5	−0.5	-	-	−0.9	-
4WKQ	IRE	−2.5	−0.8	−5.3	-	-	−1.7	−0.2
5P9J	8E8	−2.9	−1.9	−6.8	-	−1.0	0.1	−0.8
6MX8	6GY	−2.8	−0.9	-	-	-	-	-
1UWH	BAX	−0.2	−1.3	−2.3	−3.6	-	−0.9	0.8
3OG7	032	−2.6	−0.5	−7.2	-	-	−0.9	2.3
4AN2	EUI	−0.7	-	0.8	-	-	-	−0.9
5CSW	P06	−2.1	−1.4	−5.0	-	-	−2.4	1.6
5L2I	LQQ	−0.1	0.0	-	-	−0.1	-	−1.4
5L2S	6ZV	−2.9	−0.8	0.4	-	−0.1	-	−0.4
5L2T	6ZZ	−0.1	−1.4	-	-	−1.6	-	−1.8
Interaction Modes		CH-π, NH-π	CH-π, NH-π	H-Bond, CH-π, Cation-π, NH-π	H-Bond, CH-π	CH-π	H-Bond, CH-π, NH-π, OH-π	H-Bond, CH-π, NH-π, Ionic Int.

a. The list is sorted alphabetically according to the PDB IDs with a dividing line that separates 27 tyrosine kinases (above) and 7 serine/threonine kinases (below). b. The number in parenthesis represents generic residue ID according to the KLIFS numbering convention [26]. c. “-” indicates the given residue is not within intermolecular interaction cutoff distance (5.5 Å).

**Table 5:** Pairwise interaction energies ($∆E_{int\;}^{aq}\;\mathrm{in}\;\mathrm{kcal}/\mathrm{mol}$ ) between PKI drugs and surrounding residues in the front interface ^a^.

PDB ID	Ligand ID	L(3) ^b^	F(47)	G(51)	L(77)	F(82)
Structure Elements		β Sheet I	Hinge	Linker	β Sheet VII	DFG Motif
1IEP	STI	−1.1	−2.2	−1.0	−2.7	−3.0
1M17	AQ4	−3.9	- ^c^	4.2	−2.4	-
1XKK	FMM	−4.4	-	−0.8	−2.2	-
2GQG	1N1	−1.9	−3.5	−0.4	−1.9	-
2IVU	ZD6	−2.3	-	−1.2	−2.2	-
2JIV	HKI	−0.2	-	-	−3.4	-
2WGJ	VGH	-	-	−0.4	-	-
3AOX	EMH	2.4	-	0.3	2.4	-
3C7Q	XIN	−1.4	−3.3	−1.4	−0.5	-
3CS9	NIL	−1.4	−1.5	−1.4	−2.6	−3.6
3EYG	MI1	−0.9	−1.7	-	−0.7	-
3LXK	MI1	−0.5	-	−1.2	−3.1	-
3OXZ	0LI	−2.2	−1.3	-	−2.8	−1.0
3UE4	DB8	−1.9	−0.6	−0.9	−0.1	-
3WZD	LEV	−4.4	−2.6	−2.3	−2.8	-
3ZBF	VGH	−1.6	-	−1.4	−3.2	-
4AG8	AXI	−3.3	−3.7	−2.8	−3.0	−1.1
4AGD	B49	−2.4	−0.5	−1.3	−1.5	−0.8
4ASD	BAX	−2.1	−2.7	-	−0.9	−4.0
4G5J	0WN	−1.6	-	−1.2	−1.9	-
4I23	1C9	−1.7	-	−1.6	-	-
4MKC	4MK	−0.7	-	−2.4	−1.1	-
4U0I	0LI	−2.8	-	−1.1	−2.2	−2.0
4U5J	RXT	−2.6	-	−1.2	−3.4	-
4WKQ	IRE	−1.9	-	−1.2	−2.3	-
5P9J	8E8	0.1	-	0.0	−2.2	-
6MX8	6GY	−4.8	-	−3.2	−1.3	-
1UWH	BAX	-	-	-	-	−1.5
3OG7	032	-	-	−4.6	-	-
4AN2	EUI	−1.4	−3.6	−0.1	−0.2	−2.4
5CSW	P06	-	-	-	-	-
5L2I	LQQ	-	-	-	−0.2	-
5L2S	6ZV	-	-	-	6.1	-
5L2T	6ZZ	-	-	-	−3.7	-
Interaction Modes		CH-π	π-π, CH-π, cation-π. NH-π	H-Bond, CH-π, NH-π	CH-π	π-π, CH-π

a. The list is sorted alphabetically according to the PDB IDs with a dividing line that separates 27 tyrosine kinases (above) and 7 serine/threonine kinases (below). b. The number in parenthesis represents generic residue ID according to the KLIFS numbering convention [26]. c. “-“ indicates the given residue is not within intermolecular interaction cutoff distance (5.5 Å) of PKI drugs.

**Table 6:** Intermolecular interaction energies between lenvatinib and residues from. vascular endothelial growth factor receptor 2.

Area	Residue	Interaction Mode	$∆E_{B2PLYP\;}^{g}$(kcal/mol)	Δ*E_Deh_* (kcal/mol)	$∆E_{int\;}^{aq}$(kcal/mol) ^a^
Hinge	Cys919(48) ^b^	H-bond, SH-π, CH-π	−4.8	2.6	−2.2
Back Interface	Val848(11)	CH-π	−2.9	0.6	−2.3
	Lys868(17)	CH-π	−1.3	0.2	−1.1
	Glu885(24)	H-bond	−1.5	1.9	0.4
	Val916(36)	CH-π	−2.8	0.3	−2.4
Front Interface	Leu840(3)	CH-π	−4.3	−0.1	−4.4
	Phe918(47)	π-π, CH-π	−4.3	1.7	−2.6
	Gly922(51)	CH-π, NH-π	−3.4	1.1	−2.3
	Leu1035(77)	CH-π	−3.2	0.4	−2.8
Other	Gly841(4)	H-bond, CH-π	−0.5	−0.2	−0.7
	Cys1045(80)	SH-π, CH-π	−3.1	1.6	−1.4

a. $∆E_{int\;}^{aq}=∆E_{B2PLYP\;}^{g}+∆E_{Deh\;}^{}\;\mathrm{according}\;\mathrm{to}\;\mathrm{Equation}\;\left(2\right)$. b. The number in parenthesis represents generic residue ID according to the KLIFS numbering convention [25].

## Data Availability

Majority of data are provided in the form of Tables in the manuscript.

## Supplementary Materials

The following is available online at https://www.mdpi.com/article/10.3390/molecules27207124/s1, Table S1: List of 30 FDA-approved protein kinase drugs.
