# Supplementary material for: Molecular Recognition of FDA-Approved Small Molecule Protein Kinase Drugs in Protein Kinases
Source: Molecules. 2022 Oct 21;27(20):7124. doi: 10.3390/molecules27207124 (PMC9611543; doi:10.3390/molecules27207124)
Supplement: Supplementary file 1 [file molecules-27-07124-s001.zip › molecules-1894327-supplementary.pdf]

Table S1. List of 30 FDA-approved protein kinase drugs.

Drug	Company	Inhibitor Type	Year Aproved	Known targets	Disease
Abemaciclib	Lilly	I	2017	CDK4/6	Breast Ca
Afatinib	Boehringer Ingelheim	I	2013	EGFR, ErbB2, ErbB4	NSCLC
Alectinib	Hoffman-LaRoche	I	2015	ALK and RET	NSCLC, ALK-positive
Axitinib	Pfizer	I	2012	VEGFR1/2/3, PDGFRβ	RCC, advanced
Bosutinib	Pfizer	I	2012	BCR-Abl, Src, Lyn, and Hck	CML
Brigatinib	Ariad	I	2017	ALK, ROS1, IGF-1R, Flt3, EGFR	NSCLC, ALK-positive
Ceritinib	Novartis	I	2014	ALK, IGF-1R, InsR, ROS1	NSCLC, ALK-positive after crizotinib resistance
Cobimetinib	Genentech	III	2015	MEK1/2	Melanoma with BRAF mutations together with vemurafenib
Crizotinib	Pfizer	I	2011	ALK, MET (HGFR), ROS1, MST1R	NSCLC, ALK-positive or ROS1-positive
Dabrafenib	GSK	I	2013	B-Raf	Melanoma and NSCLC with BRAF mutations
Dacomitinib	Pfizer	I	2018	EGFR family	EGFR-mutant NSCLC
Dasatinib	Bristol-Myers Squibb	I	2006	BCR-Abl, Src, Lck, Yes, Fyn, Kit, EphA2, PDGFRβ	CML
Erlotinib	Genentech	I	2004	EGFR	NSCLC; pancreatic cancer
Gefitinib	Astra Zeneca	I	2003-2005, 2015	EGFR	NSCLC
Ibrutinib	Pharma-cyclics and J&J	I	2013	Bruton tyrosine kinase	Mantle cell lymphoma; CLL; Waldenstrom's macro-globulinemia; marginal zone lymphoma; graft vs. host disease
Imatinib	Novartis	II	2001	BCR-Abl, Kit, and PDGFR	CML and ALL, Ph+; aggressive systemic mastocytosis; CEL; DFSP; HES; GIST; MDS/MDP

Table S1. (Continued)

Drug	Company	Inhibitor Type	Year Approved	Known targets	Disease
Lapatinib	GSK	I	2007	EGFR, ErbB2	Breast cancer
Lenvatinib	Easai	I	2015	VEGFR1/2/3, PDGFR, FGFR, Kit, RET	Different-iated thyroid cancer
Neratinib	Puma	I	2017	ErbB2/HER2	HER+ breast cancer
Nilotinib	Novartis	II	2007	BCR-Abl, PDGFR, DDR1	CML, Ph+
Nintedanib	Boehringer Ingelheim	I	2014	FGFR1/2/3, PDGFRα/β, VEGFR1/2/3, Flt3	Pulmonary fibrosis, idiopathic
Palbociclib	Park Davis	II	2015	CDK4/6	Breast cancer, ER+ and HER2+
Ponatinib	Ariad	II	2012	BCR-Abl, BCR-Abl T315I, VEGFR, PDGFR, FGFR, EphR, Src family kinases, Kit, RET, Tie2, Flt3	CML or ALL, Ph+
Ribociclib	Novartis	I	2017	CDK4/6	Breast cancer
Ruxolitinib	Incyte	I	2011	JAK1/2	Myelofibrosis; polycythemia vera
Sorafenib	Onyx	II	2005	VEGFR1/2/3, B-/C-Raf, mutant B-Raf, Kit, Flt3, RET, and PDGFRß	Thyroid cancer, differentiated; hepatocellular carcinoma; RCC
Sunitinib	Pfizer	I	2006	PDGFRα/β, VEGFR1/2/3, Kit, Flt3, CSF-1R, and RET	RCC; GIST; pancreatic neuroendocrine tumors
Tofacitinib	Pfizer	I	2012	JAK3	Rheumatoid arthritis
Vandetanib	Astra-Zeneca	I	2011	EGFRs, VEGFRs, RET, Brk, Tie2, EphRs, and Src family kinases	Thyroid cancer, medullary
Vemurafenib	Genentech	I	2011	A/B/C-Raf and B-Raf (V600E)	Melanoma with the BRAFV600E mutation
